# Epidemic characteristics and transmission risk prediction of brucellosis in Xi'an city, Northwest China

**DOI:** 10.3389/fpubh.2022.926812

**Published:** 2022-07-22

**Authors:** Chenxi Zhao, Kun Liu, Chenghao Jiang, Xiao Wei, Shuxuan Song, Xubin Wu, Xiaohui Wen, Ting Fu, Li Shen, Zhongjun Shao, Qian Li

**Affiliations:** ^1^Ministry of Education Key Lab of Hazard Assessment and Control in Special Operational Environment, Department of Epidemiology, School of Public Health, Air Force Medical University, Xi'an, China; ^2^Department of Epidemiology, School of Public Health, Baotou Medical College, Baotou, China; ^3^Department of Geospatial Information Engineering, School of Remote Sensing and Information Engineering, Wuhan University, Wuhan, China; ^4^Department of Infectious Disease Control and Prevention, Xi'an Center for Disease Prevention and Control, Xi'an, China

**Keywords:** brucellosis, spatiotemporal expansion, socioeconomic factors, boosted regression tree models, risk areas

## Abstract

Human brucellosis (HB) has re-emerged in China since the mid-1990s, and exhibited an apparent geographic expansion shifted from the traditional livestock regions to the inland areas of China. It is often neglected in non-traditional epidemic areas, posing a serious threat to public health in big cities. We carried out a retrospective epidemiological study in Xi'an, the largest city in northwestern China. It utilizes long-term surveillance data on HB during 2008–2021 and investigation data during 2014–2021. A total of 1989 HB cases were reported in Xi'an, consisting of 505 local cases, i.e., those located in Xi'an and 1,484 non-local cases, i.e., those located in other cities. Significantly epidemiological heterogeneity was observed between them, mainly owing to differences in the gender, occupation, diagnostic delays, and reporting institutions. Serological investigations suggested that 59 people and 1,822 animals (sheep, cattle, and cows) tested positive for brucellosis from 2014 to 2021, with the annual average seroprevalence rates were 1.38 and 1.54%, respectively. The annual animal seroprevalence rate was positively correlated with the annual incidence of non-local HB cases. Multivariate boosted regression tree models revealed that gross domestic product, population density, length of township roads, number of farms, and nighttime lights substantially contributed to the spatial distribution of local HB. Approximately 7.84 million people inhabited the potential infection risk zones in Xi'an. Our study highlights the reemergence of HB in non-epidemic areas and provides a baseline for large and medium-sized cities to identify regions, where prevention and control efforts should be prioritized in the future.

## Introduction

Brucellosis, an infectious allergic disease caused by the members of the *Brucella* genus, is endemic in most developing countries and causes a heavy health burden worldwide (1–4). Currently, 12 animal species have been recognized as the reservoir hosts of *Brucella*, including domestic animals such as cattle, goats, sheep, and pigs (5, 6). Humans acquire infection chiefly through direct contact with the tissues or fluids of infected livestock or by consuming contaminated dairy products, including unpasteurized milk and cheeses (7). Acute infection of brucellosis presents a flu-like syndrome, accompanied with an undulating fever, headaches, muscle aches, arthralgia, fatigue, and sweats (8). These non-specific manifestations prolong the diagnostic delay time, leading to debilitating complications such as arthritis, orchitis, hepatitis, encephalomyelitis, and endocarditis (9). Although effective vaccines against brucellosis have been developed for animals, no such vaccine for application in humans is available (10). More than 500,000 new human cases are reported annually. It has also been reported that brucellosis often causes abortions in infected animals, which adversely affects livestock production (11). Hence, brucellosis remains a global threat to public health and economy with an enormous tangible and intangible cost for both individuals and the society (2, 12).

In China, brucellosis has re-emerged in recent years with a significant spatial trend. Previous studies have suggested that the disease is highly endemic among dairy herds and small ruminants in China (13, 14). In addition, 524,980 cases of human brucellosis (HB) were reported to the National Infectious Surveillance System during 2004–2018, with a significantly higher average annual incidence in 2012–2018 than that in 2004–2011 (15). Moreover, the high incidence of HB is concentrated in northern China, with an apparent shift in the geographic expansion from northern traditional livestock regions to more urban southern inland areas (16). Our previous study conducted in Shaanxi Province demonstrated similar conclusions regarding the spatiotemporal distribution of HB (17). Owing to rapid and sometimes uncontrolled urbanization, people may face closer encounters with infected animals (18). Although brucellosis is endemic, it still does not receive much attention from health officials in non-epidemic regions. As a result, it may lead to a noticeable increase in the number of HB cases in big cities (19). Xi'an, the largest city in Northwest China, with a prosperous economy and an extensive network of transport, is witnessing a rapid increase in the consumption of meat and dairy products, which is driving extensive livestock breeding, slaughtering, and trading. Even though Xi'an is a traditional non-epidemic region for brucellosis, the incidence of HB has increased 20 times in the past 10 years with sharply declining animal seroprevalence. Additionally, the cases reported from different regions exhibit apparently different epidemiological characteristics. These observations indicate that the epidemic patterns of brucellosis have changed dramatically over time in Xi'an.

Improving the understanding of the epidemiological characteristics and identifying the risk factors responsible for epidemic transmission are necessary for preventing and controlling HB. Previous studies have mainly focused on determining the local seroprevalence, demographic characteristics, and risk factors for HB infections (20–22). However, very few investigations have investigated the long-term systematic epidemiological characteristics and identified spatially driving factors of brucellosis. To devise better targeted surveillance and control efforts for HB in the future, we conducted a retrospective study in Xi'an: (1) to explore the dynamic epidemiological characteristics of local and non-local cases based on long-term surveillance data from 2008 to 2021; (2) to delineate the seroprevalence change patterns based on the incidence of local and non-local cases and the seroprevalence of occupational population and livestock by serological investigations; and (3) to evaluate the geographic heterogeneity of local HB cases, identify risk determinants, and assess potential-risk regions and population in Xi'an.

## Materials and methods

### Study area

Xi'an is the capital city of Shaanxi Province, Northwest China. It borders the major endemic regions for HB, including Inner Mongolia, Gansu, Ningxia, and Shanxi Provinces (16). Xi'an contains 13 counties and districts comprising 178 townships with an area of 10,108 km^2^, extending from 33°42'−34°45' to 107°40'−109°49', and a population of 12.95 million in 2020 (http://tjj.xa.gov.cn). Xi'an is one of the core economic clusters of the Belt and Road Initiative, which is principally intended to promote commercial associations and connectivity amongst several continents, is considered an influential trade route and indispensable logistics hub for Central, South, and West Asian countries (23). The gross domestic product (GDP) of Xi'an expanded by 5.2% year-on-year in 2020 and reached 1.002 trillion yuan, exceeding the 1 trillion yuan threshold (http://tjj.xa.gov.cn). The city is dominated by manufacturing and tourism industries. Only a small proportion of the population is engaged in agriculture and animal husbandry. It has more than 13,000 km of highways. Nine national highways intersect here, making it one of the largest nodal cities of the National Trunk Highway System of China.

### Data collection and management

#### Human brucellosis

In China, brucellosis is classified as a class B notifiable infectious disease. Human cases of brucellosis diagnosed at medical and health institutions require to report to the local Center for Disease Control and Prevention (CDC) compulsorily. HB cases are confirmed by a series of evidence, which includes contact history (such as contact with infected animals or contaminated animal products and people living in endemic areas), clinical manifestations (e.g., an undulating fever, headaches, muscle aches, arthralgia, fatigue, and sweats), and laboratory test consists of primary screening tests [such as the Rose Bengal plate test (RBPT), gold immunochromatography assay (GICA), or/and enzyme-linked immunosorbent assay (ELISA)] and confirmatory tests [such as the serum agglutination test (SAT), complement fixation test (CFT), Coomb's test, or/and isolation of *Brucella* spp.] following a standard protocol (Diagnostic Criteria for Brucellosis, WS 269-2007/WS 269-2019). Confirmatory tests will be carried out on the positive samples of preliminary screening tests. Testing positive by either correspondence method is considered preliminary screening or confirmed positive. Positive cases for animals are confirmed by the same laboratory test as HB cases.

We collected all reported HB cases from January 2008 to December 2021 from the Xi'an Information System for Disease Control and Prevention and established a database using demographic characteristics such as the identification number, name, age, sex, residential address, occupation, date of symptom onset, date of diagnosis, and reporting institution. HB cases were categorized as local or non-local, depending on whether their residential address is located in Xi'an. Farmers, herdsmen, and abattoir workers were classified as rural workers, while other occupations such as healthcare workers, students, and teachers were defined as non-rural workers. Diagnostic delays were defined as the time interval between the onset of HB symptoms noted by the patient and its confirmed diagnosis at a healthcare institution (24).

According to the Xi'an Brucellosis Prevention and Control Plan, three counties with the highest incidence in the previous year are annually selected as active surveillance regions, for conducting serological investigations on animals (sheep, cattle, and cows) and humans, especially on those with occupational exposure. Information about serological investigations conducted during 2014–2021 were collected, including active surveillance regions, number of people tested and those found positive, and the number of animals tested and those found positive.

#### Socioeconomic factors

Following previous studies and expert opinions on the topic, we identified 13 potential risk factors for HB transmission: number of farms, number of slaughterhouses, sheep density, goat density, cattle density, pig density, GDP, length of first-grade highways, length of secondary roads, length of township roads, nighttime lights, population density, and urban accessibility. We used the information collected through these risk factors to explore the relationship between the spatiotemporal distribution and spatial expansion of HB in Xi'an city. To identify the location of slaughterhouses and farms that might be involved in the spread of infection and determine their spatial distribution, we employed the web crawler technique to determine the running time (beginning and ending year of slaughterhouses and farms), address, and the species of animals raised and/or butchered at farms and slaughterhouses. Web crawler is a program or script that automatically crawls through the web to extract information according to established rules. This technique is widely used in public health (25). The farms and slaughterhouses were eligible for inclusion if they met the following criteria. (1) The study period must intersect with the running time. (2) Their addresses must be specific and detailed, reflecting the precise latitude and longitude to allow further study. (3) The species of livestock kept or slaughtered must include sheep, goats, cattle, or pigs. In addition, raster-type density data regarding sheep, goats, cattle, and pigs were extracted from the Gridded Livestock of the World (GLW v3.1) database at a spatial resolution of 5 min, which was supported by the Food and Agriculture Organization of the UN. We obtained 1 km × 1 km GDP datasets and 3 km × 3 km spatial resolution for the length of first-grade highways, secondary roads, and township roads generated by line-type information from the National Catalog Service for Geographic Information. Nighttime lights, which is an effective representation of human activities and prosperity of a city, was obtained from the United States Defense Meteorological Satellite Program's Operational Linescan System (DMSP-OLS). The DMSP-OLS light at night has 30 × 30 arc-second gridded nocturnal luminosity equivalent to 1 km. The 1 km × 1 km population density was collected from the WorldPop dataset. Travel and commercial activities, which attribute to the dissemination of *Brucella* by increasing opportunities for human exposure, may account for a much higher risk of brucellosis in highly accessible and interconnected regions than that observed in other places. Hence, we downloaded ~1 km × 1 km-gridded urban accessibility from the European Commission Joint Research Center, which demonstrates the travel time to large cities with a population of ≥50,000.

### Data analysis

#### Descriptive analysis

Address-specific monthly incidence was plotted along with annual incidence curves to display the temporal distribution of HB during 2008–2021. A radar chart of monthly incidence was generated to explore the temporal dynamics and seasonal trend of all HB cases. A thematic map was created to visualize the spatiotemporal expansion of local cases.

#### Statistical analysis

A Boosted Regression Trees (BRT) model was applied to fit the multivariate empirical relationship between the probability of HB occurrence and socioeconomic factors. BRT is a powerful approach to ecological research and has been widely used for risk mapping of infectious diseases such as avian influenza, visceral leishmaniasis, and hemorrhagic fever with renal syndrome (26–28). The BRT model has the advantages of two tree-based methods, regression trees and machine learning techniques, allows non-linear relationships with relatively high prediction performance and interaction effects among multiple variables (29).

After excluding grid cells with less than one-third of the area occupied, we assessed the relative risk of HB based on a regular grid of 1,063 3 km × 3 km cells divided from Xi'an city, among which 192 spatial coordinates were assigned to 496 local cases. The information obtained using thirteen potential socioeconomic predictors was extracted based on their locations or raster maps and was averaged across 3 km × 3 km grid cells.

The BRT modeling was carried out in sequential steps. First, we employed a “case-control” design; all grid cells reporting local cases were assigned as “cases,” while those without cases were marked as “controls.” A dataset consisting of 192 cases and 768 randomly selected controls with a 1:4 case-control ratio was established for each model. Second, the bootstrap dataset was randomly portioned into a training dataset with 75% of grid cells and a test dataset with 25% of grid cells. Third, we fitted the BRT model for each bootstrap dataset using the training dataset and validated using the test dataset. Finally, the fitted model was applied to predict the probability of HB occurrence during 2008–2021 in Xi'an. Based on the above steps, we fitted a collective of 300 BRT models to increase the robustness of the model simulations and quantify model uncertainty. Trees were built with parameters including a tree complexity of 3, a learning rate of 0.01, and a bag fraction of 0.75. Model performance was evaluated by 10-fold cross-validation that allows to testing the model against withheld portions of the data not used in model fitting. The area under the curve (AUC) was calculated to evaluate the predictive power of the model. A risk map of HB in Xi'an in 2008–2021 was then created based on the average predicted probability of 300 repetitions of each grid cell. Furthermore, we considered every grid cell with the average predicted probability above a threshold value as the risk zone for HB transmission. We also superimposed the raster-type population data and estimated population at the risk region in counties.

All analyses were conducted with R version 4.1.2. The BRT models were constructed by using the “ggBRT” and “gbm” packages and the threshold was calculated by using the “InformationValue” package. The spatial analyses were performed using ArcGIS 10.2 Software (ESRI Inc.; Redlands, CA, USA). All statistical tests were two-sided, and a *P* value < 0.05 was considered statistically significant.

### Ethical statement

The brucellosis surveillance and investigations are governmental public health task under the charge of the Xi'an Municipal CDC. Therefore, an ethical review by an ethics committee was not required.

## Results

### Epidemiological features and spatiotemporal distribution of HB

**Figure 1:**
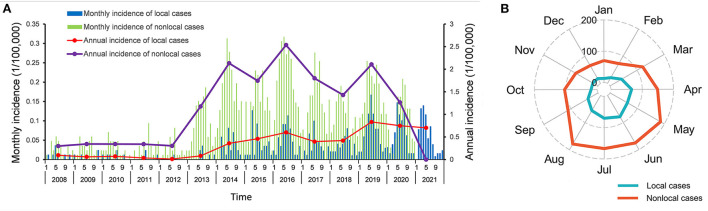
Temporal distribution and seasonal trend of human brucellosis in Xi'an, 2008–2021. **(A)** Monthly and annual incidences of local and non-local cases. **(B)** Monthly number of local and non-local cases.

A total of 505 local and 1,484 non-local HB cases were reported in Xi'an city from January 2008 to December 2021, corresponding to the annual average incidences of 0.348 and 1.217 per 100,000 persons, respectively. The epidemic persisted at a relatively low level from 2008 (33 cases, 0.197 per 100,000 persons) to 2012 (27 cases, 0.158 per 100,000 persons). For local cases, the annual incidence soared in 2013 (7 cases, 0.082 per 100,000 persons), peaked in 2019 (85 cases, 0.833 per 100,000 persons), and then remained at a relatively high level. For non-local cases, a peak was observed in 2016 (224 cases, 2.536 per 100,000 persons), followed by a sharp and consistent decline, despite fluctuations in the number of cases (Figure 1A). A similar seasonal trend for both local and non-local HB cases during 2008–2021 was observed from April to August, accounting for 66.34 and 57.75% of the total cases, respectively (Figure 1B). All HB cases showed a male-to-female ratio of 2.69. The proportion of men of 68.51% was significantly smaller in the local cases than that of 74.39% in the non-local cases (*P* = 0.01) (Table 1). The median age of all affected people was 49.00 years (Interquartile range: 34.00–57.00). There was no statistical difference in age between local and non-local cases (*P* = 0.113). In terms of occupation, more than 70% of the cases were engaged in high-risk jobs of brucellosis. The local cases had a significantly smaller proportion of rural workers of 71.09% than that of 79.92% of non-local cases (*P* < 0.001). In addition, the reported cases were mainly concentrated in the diagnostic delays group <30 days. The local cases also had significantly shorter diagnostic delays than that for non-local cases (*P* < 0.001). There were only 37 (1.86%) HB cases reported by non-CDC agencies. The proportion of local cases diagnosed at a CDC agency was 2.97%, significantly higher than that of 1.48% for non-local cases (*P* = 0.033).

**Table 1:** Epidemiological characteristics of local and non-local cases in Xi'an from 2008 to 2021.

**Characteristics**	**Reporting cases (*****n*** = **1,989)**	**Local cases (*****n*** = **505)**	**Non-local cases (*****n*** = **1,484)**	***P*** **-value**
**Gender**				
Male	1,450 (72.90%)	346 (68.51%)	1,104 (74.39%)	0.010
Female	539 (27.10%)	159 (31.49%)	380 (25.61%)	
**Age**	49.00 (34.00–57.00)	49.00 (35.50–59.00)	48.00 (33.00–57.00)	0.113
**Occupation**				
Rural workers	1,544 (77.63%)	359 (71.09%)	1,186 (79.92%)	<0.001
Non-rural workers	445 (22.37%)	146 (28.91%)	298 (20.08%)	
**Diagnostic delays**				
≤30 days	1,661 (83.51%)	443 (87.72%)	1,218 (82.07%)	<0.001
31–60 days	157 (7.89%)	34 (6.73%)	123 (8.29%)	
≥61 days	171 (8.60%)	28 (5.55%)	143 (9.64%)	
**Reporting institution**				
CDC	37 (1.86%)	15 (2.97%)	22 (1.48%)	0.033
Non-CDC	1,952 (98.14%)	490 (97.03%)	1,462 (98.52%)	

**Figure 2:**
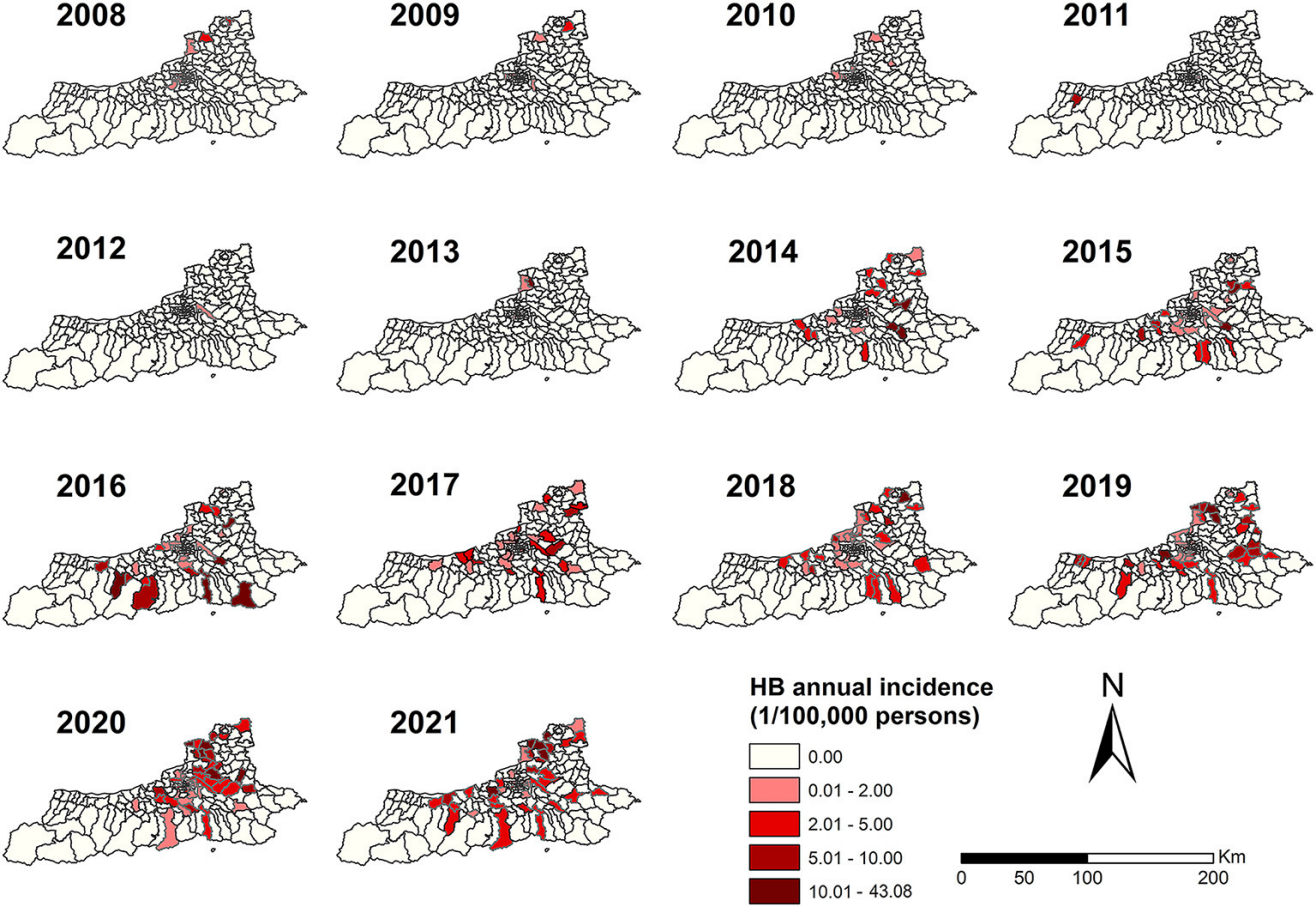
Spatial and temporal distributions of human brucellosis in Xi'an, 2008–2021.

Local HB cases were distributed mainly in the northeastern and central parts of Xi'an; they were rarely detected in the southwestern part of the region (Figure 2). From 2008 to 2013, the local HB cases were chiefly scattered in the north and central parts of Xi 'an. In addition, only 19 townships reported HB, accounting for 10.67% (19/178) of the total townships. Since 2014, brucellosis has gradually spread to the southwest of Xi'an too. Until 2021, 124 out of 178 townships in Xi'an had reported HB cases over the study period, and a gradually extending pattern was observed for the geographic extent of HB, which spanned from the northeastern and central parts of Xi'an to its southwestern part. Five townships that reported the highest average incidences of HB were Mengcun Town, Luyuan Street, ZhangBu town, Muzhai Street, and Chonghuang Street, with the average incidences of 4.59, 4.19, 3.13, 3.08, and 3.03 per 100,000 persons, respectively.

### Seroprevalence rates of human and animal brucellosis

**Figure 3:**
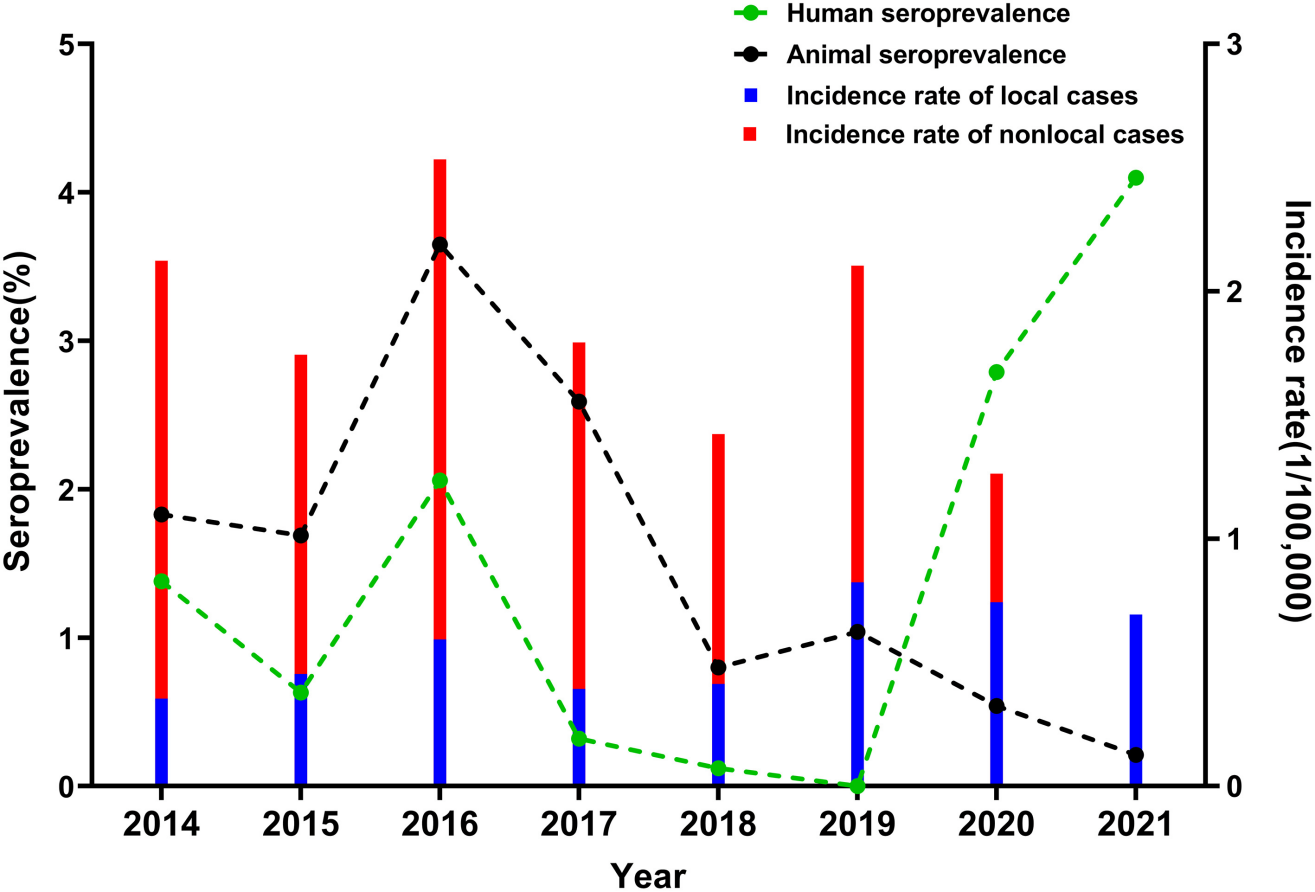
Seroprevalence of brucellosis in human and animals in Xi'an, 2014–2021. Blue and red bar charts represent the annual incidence of local and non-local cases, respectively. Green and black lines represent the annual seroprevalence of human and animals, respectively.

From 2014 to 2021, 4,263 people and 256,424 animals were sampled from six regions of Xi'an. Among these samples, 59 and 1,822 tested positive for brucellosis, with the annual average seroprevalence rates of 1.38 and 1.54%, respectively (Table 2). The human seroprevalence rate peaked in 2016 at 2.06%, steadily decreased until 2019, and then dramatically increased to 4.10% in 2021. There was no statistically significant trend for human seroprevalence rate from 2014 to 2021 (*P* for trend = 0.630). In contrast, the animal seroprevalence rates sharply declined to 0.21% in 2021 after reaching the peak in 2016 at 3.65% (*P* for trend = 0.004) (Figure 3). Notably, the cross-correlation analysis revealed that the annual animal seroprevalence rate was not significantly correlated with the incidence of local cases; instead, it was positively associated with the annual incidence of non-local cases (*r* = 0.88, *P* = 0.007) (Supplementary Figure S1). Although there seems to be a correlation between the human and animal seroprevalence rates during 2014–2018, no other significant correlations were observed among other groups during the study period.

**Table 2:** Human and animal seroprevalence rates from serological investigations according to the Xi'an Brucellosis Prevention and Control Plan from 2014 to 2021.

**Sampling year**	**Active surveillance regions**	**Humans tested**	**No. of positive cases**	**Human seroprevalence rate (%)**	**Animals tested**	**No. of positive cases**	**Animal seroprevalence rate (%)**
2014–2021		4,263	59	1.38	2,56,424	1,822	1.54
2014	Gaolin, Lintong, and Zhouzhi	362	5	1.38	6,927	127	1.83
2015	Gaolin, Lintong, and Zhouzhi	317	2	0.63	8,519	144	1.69
2016	Gaolin, Lintong, and Chang'an	340	7	2.06	7,011	256	3.65
2017	Gaolin, Huyi, and Chang'an	309	1	0.32	11,834	307	2.59
2018	Huyi, Zhouzhi, and Chang'an	815	1	0.12	16,406	131	0.80
2019	Huyi, Zhouzhi, and Xixian new area	830	0	0	12,294	128	1.04
2020	Gaolin, Chang'an, and Xixian new area	753	21	2.79	1,00,164	536	0.54
2021	Gaolin, Chang'an, and Xixian new area	537	22	4.10	93,269	193	0.21

### Identification of risk factors and prediction of high-risk areas

**Figure 4:**
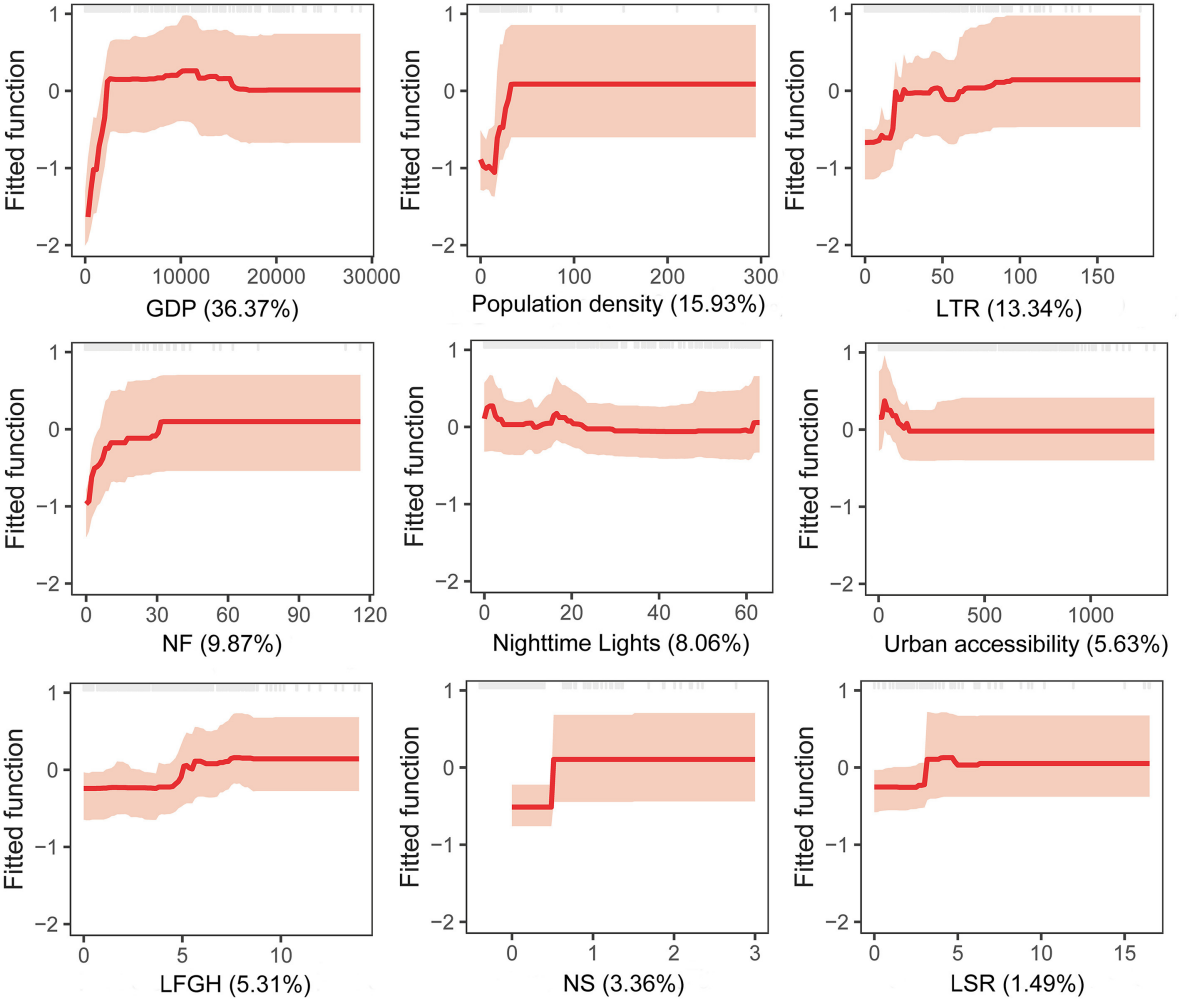
Partial dependency plots with 95% confidence intervals for nine variables. The graphs show the effect of a given predictor on the probability of occurrence of human brucellosis, while keeping all other variables at their mean. Average relative influence of each predictor is reported between parentheses. Gray tick marks across the top of each plot indicate observed data points. GDP, gross domestic product; LTR, length of township roads; NF, number of farms; LFGH, length of first-grade highways; NS, number of slaughterhouses; LSR, length of secondary roads.

**Figure 5:**
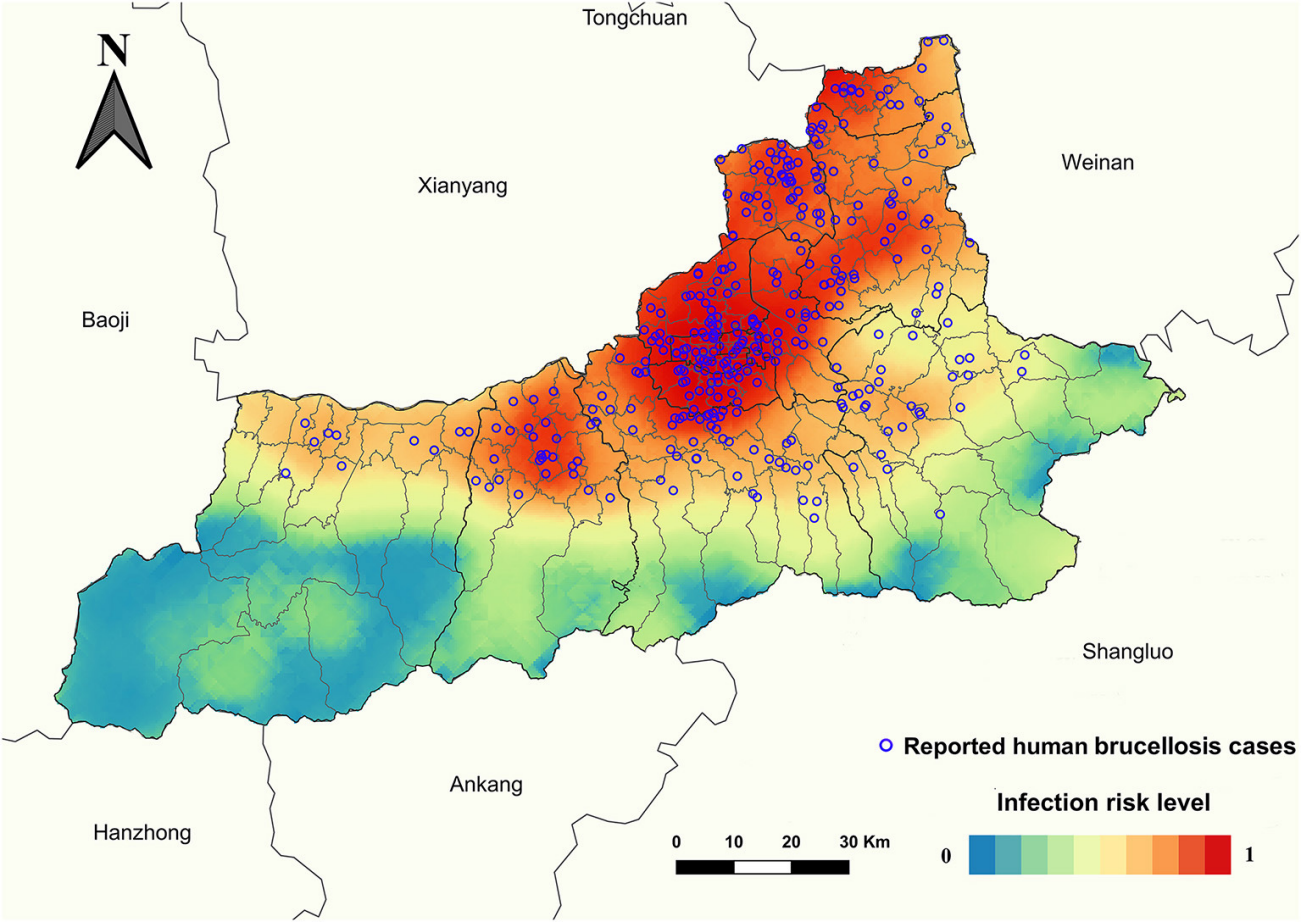
Predicted risk map of human brucellosis in Xi'an. The map was created using ArcGIS 10.2 software, ESRI Inc., Redlands, CA, USA, by the kriging interpolation method.

Grid cells with and without HB spatial coordinates showed a statistical difference (*P* < 0.001) depending on the number of slaughterhouses, number of farms, GDP, nighttime lights, population density, length of first-grade highways, length of secondary roads, length of township roads, and urban accessibility (Table 3). BRT multivariate models showed that the geographic distribution of HB is significantly associated with five factors: GDP (36.37% relative contribution), population density (15.93%), length of township roads (13.34%), number of farms (9.87%), and nighttime lights (8.06%), with an average relative contribution of collective models above the threshold of significance (100/number of predictors included in the model) of 7.69% (Table 4). The model-fitted partial dependence functions and standard deviations are plotted for nine predictors in Figure 4, and a non-linear relationship was observed between the predictors and HB occurrence. The probability of HB occurrence first increased significantly and then plateaued in response to an increase in GDP, population density, length of township roads, and the number of farms. As the nighttime lights increased, the probability of HB occurrence slightly increased at first, and then gradually decreased and plateaued, despite some fluctuations. The collective BRT models provided a high predictive accuracy based on validation statistics within Xi'an (training data AUC = 0.94, 10-fold cross-validation AUC = 0.88, test data AUC = 0.77) (Supplementary Table S1). The forecasted high endemic areas mainly occurred in the northeastern and central areas of Xi'an and overlapped well with the observed HB cases (Figure 5). A threshold value of 0.26 was adopted to convert the probability of HB occurrence into presence/absence of every grid cell (Supplementary Table S2). Integration of the gridded risk population at the county level helped estimate that 7.84 million people lived in areas at potential risk of HB in Xi'an, and most (59.31%) of them distributed in five counties (Beilin, Xincheng, Yanta, Weiyang, and Chang'an) (Supplementary Table S3).

**Table 3:** Geographic heterogeneity of socioeconomic factors for grid cells, with and without HB spatial coordinates.

**Characteristics**	**Overall (*N* = 1,063)**	**Grid cells with HB spatial coordinates (*N* = 192)**	**Grid cells without HB spatial coordinates (*N* = 871)**	***P*** **-value**
Number of slaughterhouses	0.01 (0.00–0.01)	0.01 (0.00–0.01)	0.01 (0.00–0.01)	<0.001
Number of farms	0.01 (0.00–1.00)	3.00 (0.00–8.75)	0.01 (0.00–0.01)	<0.001
Sheep density	0.01 (0.00–0.01)	0.01 (0.00–0.01)	0.01 (0.00–0.01)	0.742
Goat density	0.01 (0.00–0.01)	0.01 (0.00–0.01)	0.01 (0.00–0.01)	0.672
Cattle density	0.01 (0.00–0.01)	0.01 (0.00–0.01)	0.01 (0.00–0.01)	0.404
Pig density	0.01 (0.00–0.01)	0.01 (0.00–0.01)	0.01 (0.00–0.01)	0.575
GDP	534.89 (87.50–2,409.09)	3,777.15 (2,042.34–11,202.74)	199.95 (65.04–1,671.05)	<0.001
Nighttime lights	5.75 (0.00–19.57)	30.42 (14.29–60.83)	0.01 (0.00–12.92)	<0.001
Population density	1.39 (0.08–5.77)	7.11 (4.53–19.14)	0.44 (0.06–4.57)	<0.001
Length of first-grade highways	0.01 (0.00–3.81)	4.13 (1.82–7.37)	0.01 (0.00–3.54)	<0.001
Length of secondary roads	0.01 (0.00–0.01)	0.01 (0.00–0.01)	0.01 (0.00–0.01)	<0.001
Length of township roads	8.48 (0.00–26.11)	33.81 (20.25–62.75)	4.96 (0.00–17.45)	<0.001
Urban accessibility	143.08 (52.07–447.78)	46.19 (30.48–46.19)	210.56 (75.36–539.00)	<0.001

**Table 4:** Summary of average relative contributions (%) of predictor variables in the BRT models.

**Characteristics**	**Average relative contributions (SD), %**
GDP*	36.37 (6.01)
Population density*	15.93 (2.91)
Length of township roads*	13.34 (4.88)
Number of farms*	9.87 (2.20)
Nighttime lights*	8.06 (3.35)
Urban accessibility	5.63 (2.87)
Length of first-grade highways	5.31 (1.75)
Number of slaughterhouses	3.36 (1.39)
Length of secondary roads	1.49 (0.87)
Cattle density	0.30 (0.31)
Pig density	0.16 (0.19)
Sheep density	0.11 (0.20)
Goat density	0.07 (0.09)

* Variables with a relative contribution of >7.69 in BRT models were considered to be significantly contributing to the occurrence of human infection brucellosis. SD, Standard Deviation.

## Discussion

We utilized a longitudinal disease dataset spanning 14 years and comprehensively assessed dynamic epidemic changes in brucellosis in Xi'an city. Our study marked the strong occupational, seasonal and sexual characteristics of brucellosis, which were consistent with other studies (15, 17, 30). The primary results suggested that the local cases were mainly distributed in the northeastern and central regions of Xi'an, although we also noted a gradual spread of the geographic extent of HB southward over the study period. Animal seroprevalence was positively associated with the incidence of non-local cases. Five socioeconomic factors contributed substantially to the spatial distribution of HB in Xi'an. Moreover, we constructed a high-resolution risk map, which indicated that 7.84 million people lived in potential-risk areas of brucellosis.

We identified differences in demographic characteristics between local and non-local cases, including gender, occupation, diagnostic delays, and reporting institution. Several previous works have shown that male and rural workers were the most affected groups (31, 32). This is because men are more likely to engage in high-risk jobs that increase their exposure to brucellosis, such as livestock breeding and abattoir work. Most rural workers have no formal education and hence no knowledge on HB. As a result, they tend to indulge in behaviors that put them more at risk to brucellosis transmission from animals to humans (33, 34). In addition, the situation changed for adult population, as in some regions, women formed a major proportion of the working labor and took most of the rural work (35). Use of unpasteurized dairy products and/or contaminated tissues on a regular basis might be responsible for food-borne transmission of brucellosis (34). We also found that diagnostic delays were much shorter for local cases than that for non-local cases. It is postulated that non-local cases were misdiagnosed in cities with poor medical services owing to the non-specific manifestations of the disease. By the time they reached Xi'an for their diagnosis and treatment, they might have already been infected for a long time. However, owing to restrictions on human mobility in response to the ongoing pandemic of the coronavirus disease 2019 (COVID-19), the number of non-local cases dramatically decreased between 2020 and 2021. Restrictions and screening of travelers, especially with fever and similar symptoms, likely reduced the number of non-local cases visiting Xi'an for treatment (36). Additionally, shorter HB-diagnostic delays were observed at CDC agencies probably because of their better laboratory testing capabilities, which underlies their significance in the prevention and control of infectious diseases (24).

To investigate the infection status and understand the prevention and control effects, three counties with the highest HB incidence in the previous year were marked as active surveillance regions. In these counties, in addition to conducting serological investigations of occupationally exposed population and livestock, the CDC staff should also provide brucellosis-related information to the local population. Once an infected animal is identified, its herd should be immediately culled and serological tests should be carried out for the surrounding high-risk population. Besides the effects of active surveillance regions, the sharply declined seroprevalence indicated that extensive education and control measures enable successful containment of the spread of infection. However, frequent outbreaks may continue to occur in areas under active surveillance for a short time only, such as Xixian New Area. Therefore, it is of practical significance to improve the health education of the high-risk population in new active surveillance regions. In zoonotic infectious diseases, pathogens mostly originate from animal reservoirs, which then transmit the infection to humans. Surprisingly, animal seroprevalence was not significantly correlated with the incidence of local cases, although it was positively associated with the incidence of non-local cases. Concerning the epidemiological heterogeneity between the local and non-local cases, we speculate that the majority of non-local cases acquired infection in other cities but sought medical treatment in Xi'an. Additionally, human infection in Xi'an was probably affected by infected animals from other cities, especially from traditional epidemic regions.

Our BRT models revealed that GDP and population density were the major drivers responsible for the geographic variation of HB. Previous studies have demonstrated that changes in socioeconomic conditions, such as growth in GDP and population density, may increase the possibility of emergence of infection and its transmission (37). Over the past 14 years, the economy of Xi'an has seen rapid growth, and the city's per capita GDP has increased from 3,992 USD in 2008 to 11,476 USD in 2020. In addition, the proportion of urban population in Xi'an is continuously increasing according to Xi'an Bureau of Statistics. This increase in the living standards of Xi'an has stimulated the demand for animal protein, which, in turn, has led to an increase in livestock farming, slaughtering, and meat transportation. As a result, the public's exposure to vectors and animal hosts has increased (38). Nighttime lights, another indicator of urbanization, is also consistent with our results. A previous study demonstrated that open animal and animal-product markets may contribute to pathogen transmission (39). From 2014 to 2019, a total of 67,259 infected animals were reported in four endemic provinces around Xi'an, where the HB incidences were all at the highest levels in China (Supplementary Table S4). Improved road network facilitates easy transportation of infected animals and contaminated animal products to traditional non-epidemic regions. Township roads may also serve as a significant way of animal trading, further contributing to spatial distribution of the disease. Similarly, the number of farms plays an important role in brucellosis infections. Therefore, it is critical to follow strict quarantine measures and supervise livestock transportation and sales between cities, especially between counties and towns (40). Notably, in epidemic province, regions with lower GDP per capita and larger number of sheep and cattle are more likely to be potential risk areas (41). Different risk factors in epidemic and non-epidemic areas suggest that effective formulation of prevention and control strategies for brucellosis requires adjustments according to local conditions.

Overall, we predict that over 7.84 million people lived in potential-risk areas for brucellosis. Similar to other zoonotic diseases as the occupation largely determines the exposure to the vectors and animal hosts of brucellosis, it is unlikely that all people living in the highest-risk areas will develop the infection (42). The estimates are intended to be used as an indicator of the total number of individuals who may acquire infection through contact with infected animals or through food-borne transmission, which may help in prioritizing the investigation and control (43). Animals and dairy products in different regions displayed great variety in positive rate of brucellosis, even within the same city (44, 45). Importantly, spatiotemporal heterogeneity of HB among counties highlights the vital role of targeted surveillance for planning and timely action toward its control. People in these high-risk regions, especially male rural workers, should be the focus of efforts to increase their awareness and design guidelines for mitigating personal risk of developing infection.

Our study has a few limitations as well. Firstly, although the data quality from the Xi'an Information System for Disease Control and Prevention is expected to be highly credible, underreporting of cases may still occur because of mild and/or unnoticeable clinical symptoms. Secondly, the socioeconomic variables used for the modeling of brucellosis might change over a long period such as the study period used herein. Hence, the conclusions reached at may not be appropriate under the current period. Third, some relevant risk factors that could refine our exploration were not available to us, including, but not limited to, raster-type livestock trade volume, number of health institutions, and the amount of meat consumption. These limitations should be addressed in better-designed future studies.

## Conclusions

Our study is a comprehensive analysis of dynamic changes related to brucellosis. It evaluated the contribution of each socioeconomic determinant and identified high-risk areas and populations for the first time. Our findings highlight the prevalence of HB in non-epidemic areas and provide a baseline for large and medium-sized cities to identify regions, where prevention and control efforts should be prioritized in the future.

## Data availability statement

The original contributions presented in the study are included in the article/supplementary materials, further inquiries can be directed to the corresponding authors.

## Author contributions

ZS, KL, and CZ: concept and design. KL, QL, CZ, SS, XWu, XWen, and CJ: acquisition and analysis of data. CZ, KL, and XWei: statistical analysis. CZ, KL, and TF: drafting of manuscript. ZS, KL, QL, LS, and CZ: revision of the manuscript. All authors have approved the final version of the manuscript.

## Funding

This work was supported by the National Natural Science Foundation of China (Grant Nos. 81803289 and 42071368), the Natural Science Foundation of Shaanxi Province (Grant No. 2020JM-329), and the Key Research and Development Program of Shaanxi Provincial [Grant No. 2020SF-107(QL)].

## Conflict of interest

The authors declare that the research was conducted in the absence of any commercial or financial relationships that could be construed as a potential conflict of interest.

## Publisher's note

All claims expressed in this article are solely those of the authors and do not necessarily represent those of their affiliated organizations, or those of the publisher, the editors and the reviewers. Any product that may be evaluated in this article, or claim that may be made by its manufacturer, is not guaranteed or endorsed by the publisher.

## Abbreviations

HB: human brucellosis
GDP: gross domestic product
CDC: Center for Disease Control and Prevention
RBPT: rose bengal plate test
GICA: gold immunochromatography assay
ELISA: enzyme linked immunosorbent assay
SAT: serum agglutination test
CFT: complement fixation test
DMSP-OLS: Defense Meteorological Satellite Program Satellites
BRT: boosted regression trees
AUC: area under the curve
IQR: interquartile range
COVID-19: coronavirus disease 2019.
