# Supplementary material for: Epidemic characteristics and transmission risk prediction of brucellosis in Xi'an city, Northwest China
Source: Front Public Health. 2022 Jul 22;10:926812. doi: 10.3389/fpubh.2022.926812 (PMC9355750; doi:10.3389/fpubh.2022.926812)
Supplement: Supplementary file 1 [file Data_Sheet_1.PDF]

Supplementary materials

1. Supplementary Figures and Tables

1.1 Supplementary Tables

Table S1. Summary of average performances of collective BRT models.

**Total deviance**	1.00
**Residual deviance**	0.51
**Correlation**	0.73
**AUC**	0.94
**Deviance explained (%)**	48.77
**CV deviance**	0.66
**CV correlation**	0.58
**CV AUC**	0.88
**CV deviance explained (%)**	33.45
**Test AUC**	0.77

Table S2. The predicted presence/absence of HB across 3 km × 3 km grid cells divided from Xi'an city. A threshold value of 0.26 was adopted to convert the probabilities of HB occurrence into presence/absence. Object ID represents the identity of every grid cell.

Object ID	Probability of HB occurrence	Presence/absence
1	0.024	Absence
2	0.031	Absence
3	0.173	Absence
4	0.254	Absence
5	0.271	Presence
6	0.028	Absence
7	0.024	Absence
8	0.032	Absence
9	0.035	Absence
10	0.024	Absence
11	0.489	Presence
12	0.024	Absence
13	0.860	Presence
14	0.035	Absence
15	0.028	Absence
16	0.056	Absence
17	0.027	Absence
18	0.024	Absence
19	0.024	Absence
20	0.024	Absence
21	0.135	Absence
22	0.024	Absence
23	0.025	Absence
24	0.275	Presence
25	0.188	Absence
26	0.556	Presence
27	0.024	Absence
28	0.453	Presence
29	0.378	Presence
30	0.035	Absence
31	0.024	Absence
32	0.406	Presence
33	0.025	Absence
34	0.024	Absence
35	0.027	Absence
36	0.026	Absence
37	0.289	Presence
38	0.100	Absence
39	0.590	Presence
40	0.060	Absence
41	0.217	Absence

42	0.024	Absence
43	0.552	Presence
44	0.026	Absence
45	0.166	Absence
46	0.067	Absence
47	0.024	Absence
48	0.740	Presence
49	0.404	Presence
50	0.024	Absence
51	0.024	Absence
52	0.051	Absence
53	0.477	Presence
54	0.214	Absence
55	0.905	Presence
56	0.364	Presence
57	0.870	Presence
58	0.026	Absence
59	0.611	Presence
60	0.030	Absence
61	0.403	Presence
62	0.249	Absence
63	0.204	Absence
64	0.038	Absence
65	0.642	Presence
66	0.024	Absence
67	0.600	Presence
68	0.024	Absence
69	0.091	Absence
70	0.029	Absence
71	0.725	Presence
72	0.027	Absence
73	0.317	Presence
74	0.024	Absence
75	0.025	Absence
76	0.052	Absence
77	0.025	Absence
78	0.250	Absence
79	0.024	Absence
80	0.024	Absence
81	0.088	Absence
82	0.025	Absence
83	0.031	Absence
84	0.608	Presence
85	0.024	Absence
86	0.024	Absence
87	0.025	Absence
88	0.448	Presence
89	0.025	Absence

90	0.024	Absence
91	0.024	Absence
92	0.342	Presence
93	0.270	Presence
94	0.024	Absence
95	0.059	Absence
96	0.572	Presence
97	0.025	Absence
98	0.250	Absence
99	0.459	Presence
100	0.024	Absence
101	0.024	Absence
102	0.027	Absence
103	0.024	Absence
104	0.173	Absence
105	0.859	Presence
106	0.666	Presence
107	0.086	Absence
108	0.026	Absence
109	0.028	Absence
110	0.024	Absence
111	0.059	Absence
112	0.030	Absence
113	0.229	Absence
114	0.024	Absence
115	0.085	Absence
116	0.024	Absence
117	0.331	Presence
118	0.024	Absence
119	0.024	Absence
120	0.290	Presence
121	0.445	Presence
122	0.497	Presence
123	0.038	Absence
124	0.175	Absence
125	0.074	Absence
126	0.099	Absence
127	0.529	Presence
128	0.121	Absence
129	0.024	Absence
130	0.025	Absence
131	0.034	Absence
132	0.051	Absence
133	0.024	Absence
134	0.292	Presence
135	0.028	Absence
136	0.077	Absence
137	0.719	Presence

138	0.386	Presence
139	0.036	Absence
140	0.365	Presence
141	0.426	Presence
142	0.027	Absence
143	0.024	Absence
144	0.895	Presence
145	0.031	Absence
146	0.024	Absence
147	0.024	Absence
148	0.432	Presence
149	0.024	Absence
150	0.064	Absence
151	0.283	Presence
152	0.024	Absence
153	0.112	Absence
154	0.188	Absence
155	0.024	Absence
156	0.326	Presence
157	0.895	Presence
158	0.206	Absence
159	0.379	Presence
160	0.074	Absence
161	0.523	Presence
162	0.034	Absence
163	0.024	Absence
164	0.722	Presence
165	0.024	Absence
166	0.486	Presence
167	0.169	Absence
168	0.024	Absence
169	0.032	Absence
170	0.027	Absence
171	0.024	Absence
172	0.024	Absence
173	0.622	Presence
174	0.303	Presence
175	0.042	Absence
176	0.024	Absence
177	0.024	Absence
178	0.876	Presence
179	0.308	Presence
180	0.024	Absence
181	0.873	Presence
182	0.025	Absence
183	0.144	Absence
184	0.024	Absence
185	0.187	Absence

186	0.126	Absence
187	0.041	Absence
188	0.024	Absence
189	0.024	Absence
190	0.089	Absence
191	0.053	Absence
192	0.379	Presence
193	0.024	Absence
194	0.232	Absence
195	0.024	Absence
196	0.135	Absence
197	0.024	Absence
198	0.026	Absence
199	0.024	Absence
200	0.024	Absence
201	0.071	Absence
202	0.024	Absence
203	0.024	Absence
204	0.024	Absence
205	0.338	Presence
206	0.025	Absence
207	0.263	Presence
208	0.063	Absence
209	0.119	Absence
210	0.101	Absence
211	0.770	Presence
212	0.026	Absence
213	0.024	Absence
214	0.895	Presence
215	0.494	Presence
216	0.155	Absence
217	0.672	Presence
218	0.024	Absence
219	0.024	Absence
220	0.294	Presence
221	0.041	Absence
222	0.029	Absence
223	0.024	Absence
224	0.315	Presence
225	0.233	Absence
226	0.331	Presence
227	0.886	Presence
228	0.064	Absence
229	0.026	Absence
230	0.029	Absence
231	0.028	Absence
232	0.224	Absence
233	0.038	Absence

234	0.235	Absence
235	0.274	Presence
236	0.024	Absence
237	0.024	Absence
238	0.025	Absence
239	0.477	Presence
240	0.025	Absence
241	0.712	Presence
242	0.148	Absence
243	0.024	Absence
244	0.024	Absence
245	0.034	Absence
246	0.565	Presence
247	0.048	Absence
248	0.024	Absence
249	0.026	Absence
250	0.324	Presence
251	0.024	Absence
252	0.197	Absence
253	0.094	Absence
254	0.024	Absence
255	0.024	Absence
256	0.567	Presence
257	0.305	Presence
258	0.024	Absence
259	0.314	Presence
260	0.361	Presence
261	0.427	Presence
262	0.024	Absence
263	0.024	Absence
264	0.025	Absence
265	0.192	Absence
266	0.031	Absence
267	0.037	Absence
268	0.029	Absence
269	0.025	Absence
270	0.066	Absence
271	0.024	Absence
272	0.868	Presence
273	0.307	Presence
274	0.143	Absence
275	0.339	Presence
276	0.146	Absence
277	0.548	Presence
278	0.024	Absence
279	0.024	Absence
280	0.849	Presence
281	0.024	Absence

282	0.347	Presence
283	0.026	Absence
284	0.110	Absence
285	0.031	Absence
286	0.421	Presence
287	0.262	Presence
288	0.253	Absence
289	0.024	Absence
290	0.311	Presence
291	0.490	Presence
292	0.024	Absence
293	0.264	Presence
294	0.879	Presence
295	0.188	Absence
296	0.426	Presence
297	0.024	Absence
298	0.859	Presence
299	0.025	Absence
300	0.032	Absence
301	0.149	Absence
302	0.024	Absence
303	0.024	Absence
304	0.220	Absence
305	0.850	Presence
306	0.421	Presence
307	0.180	Absence
308	0.879	Presence
309	0.879	Presence
310	0.402	Presence
311	0.048	Absence
312	0.200	Absence
313	0.645	Presence
314	0.828	Presence
315	0.062	Absence
316	0.202	Absence
317	0.278	Presence
318	0.024	Absence
319	0.528	Presence
320	0.332	Presence
321	0.024	Absence
322	0.025	Absence
323	0.046	Absence
324	0.024	Absence
325	0.144	Absence
326	0.027	Absence
327	0.024	Absence
328	0.023	Absence
329	0.024	Absence

330	0.590	Presence
331	0.400	Presence
332	0.024	Absence
333	0.024	Absence
334	0.024	Absence
335	0.582	Presence
336	0.024	Absence
337	0.024	Absence
338	0.024	Absence
339	0.031	Absence
340	0.511	Presence
341	0.024	Absence
342	0.024	Absence
343	0.037	Absence
344	0.306	Presence
345	0.503	Presence
346	0.679	Presence
347	0.255	Absence
348	0.074	Absence
349	0.024	Absence
350	0.029	Absence
351	0.026	Absence
352	0.286	Presence
353	0.167	Absence
354	0.692	Presence
355	0.049	Absence
356	0.024	Absence
357	0.024	Absence
358	0.268	Presence
359	0.026	Absence
360	0.024	Absence
361	0.311	Presence
362	0.183	Absence
363	0.028	Absence
364	0.135	Absence
365	0.024	Absence
366	0.882	Presence
367	0.210	Absence
368	0.025	Absence
369	0.131	Absence
370	0.055	Absence
371	0.024	Absence
372	0.131	Absence
373	0.041	Absence
374	0.024	Absence
375	0.546	Presence
376	0.261	Presence
377	0.025	Absence

378	0.841	Presence
379	0.042	Absence
380	0.024	Absence
381	0.860	Presence
382	0.464	Presence
383	0.141	Absence
384	0.036	Absence
385	0.024	Absence
386	0.141	Absence
387	0.024	Absence
388	0.024	Absence
389	0.024	Absence
390	0.031	Absence
391	0.255	Absence
392	0.174	Absence
393	0.157	Absence
394	0.026	Absence
395	0.217	Absence
396	0.024	Absence
397	0.024	Absence
398	0.182	Absence
399	0.025	Absence
400	0.024	Absence
401	0.521	Presence
402	0.024	Absence
403	0.455	Presence
404	0.134	Absence
405	0.024	Absence
406	0.024	Absence
407	0.026	Absence
408	0.024	Absence
409	0.579	Presence
410	0.434	Presence
411	0.289	Presence
412	0.131	Absence
413	0.035	Absence
414	0.024	Absence
415	0.321	Presence
416	0.024	Absence
417	0.035	Absence
418	0.024	Absence
419	0.297	Presence
420	0.024	Absence
421	0.026	Absence
422	0.024	Absence
423	0.025	Absence
424	0.029	Absence
425	0.509	Presence

426	0.662	Presence
427	0.140	Absence
428	0.284	Presence
429	0.065	Absence
430	0.125	Absence
431	0.043	Absence
432	0.897	Presence
433	0.106	Absence
434	0.024	Absence
435	0.204	Absence
436	0.172	Absence
437	0.026	Absence
438	0.024	Absence
439	0.486	Presence
440	0.024	Absence
441	0.023	Absence
442	0.025	Absence
443	0.616	Presence
444	0.472	Presence
445	0.068	Absence
446	0.696	Presence
447	0.024	Absence
448	0.029	Absence
449	0.024	Absence
450	0.024	Absence
451	0.219	Absence
452	0.024	Absence
453	0.024	Absence
454	0.334	Presence
455	0.342	Presence
456	0.042	Absence
457	0.100	Absence
458	0.061	Absence
459	0.370	Presence
460	0.024	Absence
461	0.028	Absence
462	0.024	Absence
463	0.499	Presence
464	0.035	Absence
465	0.024	Absence
466	0.033	Absence
467	0.122	Absence
468	0.023	Absence
469	0.030	Absence
470	0.024	Absence
471	0.029	Absence
472	0.408	Presence
473	0.179	Absence

474	0.024	Absence
475	0.501	Presence
476	0.026	Absence
477	0.870	Presence
478	0.024	Absence
479	0.053	Absence
480	0.024	Absence
481	0.548	Presence
482	0.078	Absence
483	0.024	Absence
484	0.112	Absence
485	0.446	Presence
486	0.066	Absence
487	0.029	Absence
488	0.042	Absence
489	0.024	Absence
490	0.412	Presence
491	0.024	Absence
492	0.024	Absence
493	0.367	Presence
494	0.418	Presence
495	0.211	Absence
496	0.323	Presence
497	0.260	Absence
498	0.049	Absence
499	0.024	Absence
500	0.024	Absence
501	0.030	Absence
502	0.612	Presence
503	0.024	Absence
504	0.417	Presence
505	0.025	Absence
506	0.252	Absence
507	0.388	Presence
508	0.024	Absence
509	0.026	Absence
510	0.214	Absence
511	0.024	Absence
512	0.049	Absence
513	0.036	Absence
514	0.153	Absence
515	0.024	Absence
516	0.700	Presence
517	0.024	Absence
518	0.037	Absence
519	0.304	Presence
520	0.024	Absence
521	0.309	Presence

522	0.035	Absence
523	0.595	Presence
524	0.024	Absence
525	0.459	Presence
526	0.637	Presence
527	0.025	Absence
528	0.478	Presence
529	0.214	Absence
530	0.024	Absence
531	0.203	Absence
532	0.424	Presence
533	0.361	Presence
534	0.029	Absence
535	0.701	Presence
536	0.219	Absence
537	0.209	Absence
538	0.024	Absence
539	0.024	Absence
540	0.024	Absence
541	0.025	Absence
542	0.086	Absence
543	0.025	Absence
544	0.261	Presence
545	0.161	Absence
546	0.883	Presence
547	0.024	Absence
548	0.026	Absence
549	0.024	Absence
550	0.257	Absence
551	0.024	Absence
552	0.025	Absence
553	0.024	Absence
554	0.031	Absence
555	0.024	Absence
556	0.340	Presence
557	0.067	Absence
558	0.024	Absence
559	0.024	Absence
560	0.032	Absence
561	0.357	Presence
562	0.024	Absence
563	0.024	Absence
564	0.389	Presence
565	0.161	Absence
566	0.024	Absence
567	0.026	Absence
568	0.120	Absence
569	0.171	Absence

570	0.134	Absence
571	0.222	Absence
572	0.854	Presence
573	0.026	Absence
574	0.711	Presence
575	0.301	Presence
576	0.143	Absence
577	0.024	Absence
578	0.431	Presence
579	0.270	Presence
580	0.024	Absence
581	0.647	Presence
582	0.396	Presence
583	0.354	Presence
584	0.521	Presence
585	0.049	Absence
586	0.041	Absence
587	0.236	Absence
588	0.042	Absence
589	0.024	Absence
590	0.214	Absence
591	0.196	Absence
592	0.024	Absence
593	0.891	Presence
594	0.242	Absence
595	0.173	Absence
596	0.353	Presence
597	0.246	Absence
598	0.219	Absence
599	0.063	Absence
600	0.026	Absence
601	0.028	Absence
602	0.234	Absence
603	0.024	Absence
604	0.035	Absence
605	0.024	Absence
606	0.175	Absence
607	0.863	Presence
608	0.024	Absence
609	0.297	Presence
610	0.635	Presence
611	0.265	Presence
612	0.039	Absence
613	0.107	Absence
614	0.279	Presence
615	0.615	Presence
616	0.024	Absence
617	0.024	Absence

618	0.024	Absence
619	0.024	Absence
620	0.514	Presence
621	0.025	Absence
622	0.045	Absence
623	0.024	Absence
624	0.024	Absence
625	0.452	Presence
626	0.026	Absence
627	0.030	Absence
628	0.109	Absence
629	0.390	Presence
630	0.024	Absence
631	0.026	Absence
632	0.024	Absence
633	0.399	Presence
634	0.095	Absence
635	0.024	Absence
636	0.418	Presence
637	0.024	Absence
638	0.027	Absence
639	0.421	Presence
640	0.652	Presence
641	0.024	Absence
642	0.024	Absence
643	0.176	Absence
644	0.025	Absence
645	0.023	Absence
646	0.025	Absence
647	0.221	Absence
648	0.031	Absence
649	0.025	Absence
650	0.112	Absence
651	0.200	Absence
652	0.392	Presence
653	0.024	Absence
654	0.852	Presence
655	0.028	Absence
656	0.042	Absence
657	0.250	Absence
658	0.245	Absence
659	0.025	Absence
660	0.024	Absence
661	0.224	Absence
662	0.823	Presence
663	0.414	Presence
664	0.024	Absence
665	0.699	Presence

666	0.024	Absence
667	0.109	Absence
668	0.259	Absence
669	0.025	Absence
670	0.112	Absence
671	0.263	Presence
672	0.024	Absence
673	0.205	Absence
674	0.031	Absence
675	0.461	Presence
676	0.126	Absence
677	0.300	Presence
678	0.818	Presence
679	0.024	Absence
680	0.024	Absence
681	0.024	Absence
682	0.024	Absence
683	0.269	Presence
684	0.024	Absence
685	0.069	Absence
686	0.912	Presence
687	0.513	Presence
688	0.783	Presence
689	0.050	Absence
690	0.024	Absence
691	0.024	Absence
692	0.024	Absence
693	0.024	Absence
694	0.273	Presence
695	0.070	Absence
696	0.554	Presence
697	0.024	Absence
698	0.218	Absence
699	0.024	Absence
700	0.218	Absence
701	0.027	Absence
702	0.178	Absence
703	0.024	Absence
704	0.258	Absence
705	0.025	Absence
706	0.024	Absence
707	0.915	Presence
708	0.024	Absence
709	0.222	Absence
710	0.496	Presence
711	0.155	Absence
712	0.559	Presence
713	0.024	Absence

714	0.194	Absence
715	0.024	Absence
716	0.224	Absence
717	0.024	Absence
718	0.024	Absence
719	0.231	Absence
720	0.047	Absence
721	0.299	Presence
722	0.361	Presence
723	0.373	Presence
724	0.024	Absence
725	0.571	Presence
726	0.127	Absence
727	0.183	Absence
728	0.318	Presence
729	0.051	Absence
730	0.291	Presence
731	0.024	Absence
732	0.024	Absence
733	0.247	Absence
734	0.571	Presence
735	0.298	Presence
736	0.024	Absence
737	0.026	Absence
738	0.030	Absence
739	0.408	Presence
740	0.191	Absence
741	0.090	Absence
742	0.024	Absence
743	0.024	Absence
744	0.569	Presence
745	0.024	Absence
746	0.025	Absence
747	0.031	Absence
748	0.043	Absence
749	0.024	Absence
750	0.555	Presence
751	0.516	Presence
752	0.561	Presence
753	0.024	Absence
754	0.106	Absence
755	0.027	Absence
756	0.154	Absence
757	0.449	Presence
758	0.352	Presence
759	0.060	Absence
760	0.024	Absence
761	0.355	Presence

762	0.855	Presence
763	0.024	Absence
764	0.202	Absence
765	0.300	Presence
766	0.206	Absence
767	0.024	Absence
768	0.074	Absence
769	0.281	Presence
770	0.081	Absence
771	0.027	Absence
772	0.255	Absence
773	0.323	Presence
774	0.456	Presence
775	0.909	Presence
776	0.173	Absence
777	0.031	Absence
778	0.744	Presence
779	0.023	Absence
780	0.228	Absence
781	0.090	Absence
782	0.024	Absence
783	0.029	Absence
784	0.024	Absence
785	0.233	Absence
786	0.024	Absence
787	0.104	Absence
788	0.290	Presence
789	0.327	Presence
790	0.024	Absence
791	0.683	Presence
792	0.024	Absence
793	0.024	Absence
794	0.031	Absence
795	0.192	Absence
796	0.024	Absence
797	0.025	Absence
798	0.162	Absence
799	0.052	Absence
800	0.024	Absence
801	0.101	Absence
802	0.286	Presence
803	0.287	Presence
804	0.024	Absence
805	0.265	Presence
806	0.596	Presence
807	0.024	Absence
808	0.215	Absence
809	0.026	Absence

810	0.046	Absence
811	0.024	Absence
812	0.161	Absence
813	0.024	Absence
814	0.024	Absence
815	0.565	Presence
816	0.141	Absence
817	0.024	Absence
818	0.215	Absence
819	0.556	Presence
820	0.130	Absence
821	0.024	Absence
822	0.024	Absence
823	0.024	Absence
824	0.324	Presence
825	0.024	Absence
826	0.024	Absence
827	0.257	Absence
828	0.453	Presence
829	0.135	Absence
830	0.024	Absence
831	0.269	Presence
832	0.024	Absence
833	0.034	Absence
834	0.326	Presence
835	0.543	Presence
836	0.023	Absence
837	0.756	Presence
838	0.632	Presence
839	0.025	Absence
840	0.428	Presence
841	0.126	Absence
842	0.060	Absence
843	0.814	Presence
844	0.566	Presence
845	0.315	Presence
846	0.024	Absence
847	0.264	Presence
848	0.437	Presence
849	0.024	Absence
850	0.221	Absence
851	0.430	Presence
852	0.667	Presence
853	0.035	Absence
854	0.025	Absence
855	0.024	Absence
856	0.401	Presence
857	0.024	Absence

858	0.432	Presence
859	0.056	Absence
860	0.025	Absence
861	0.215	Absence
862	0.042	Absence
863	0.122	Absence
864	0.024	Absence
865	0.167	Absence
866	0.024	Absence
867	0.040	Absence
868	0.106	Absence
869	0.745	Presence
870	0.035	Absence
871	0.024	Absence
872	0.027	Absence
873	0.038	Absence
874	0.366	Presence
875	0.665	Presence
876	0.024	Absence
877	0.024	Absence
878	0.483	Presence
879	0.926	Presence
880	0.663	Presence
881	0.601	Presence
882	0.024	Absence
883	0.024	Absence
884	0.024	Absence
885	0.195	Absence
886	0.442	Presence
887	0.389	Presence
888	0.211	Absence
889	0.472	Presence
890	0.419	Presence
891	0.024	Absence
892	0.060	Absence
893	0.034	Absence
894	0.289	Presence
895	0.024	Absence
896	0.611	Presence
897	0.024	Absence
898	0.026	Absence
899	0.024	Absence
900	0.024	Absence
901	0.024	Absence
902	0.349	Presence
903	0.312	Presence
904	0.024	Absence
905	0.024	Absence

906	0.035	Absence
907	0.025	Absence
908	0.024	Absence
909	0.025	Absence
910	0.809	Presence
911	0.024	Absence
912	0.217	Absence
913	0.153	Absence
914	0.142	Absence
915	0.024	Absence
916	0.107	Absence
917	0.024	Absence
918	0.036	Absence
919	0.024	Absence
920	0.040	Absence
921	0.027	Absence
922	0.024	Absence
923	0.192	Absence
924	0.151	Absence
925	0.033	Absence
926	0.086	Absence
927	0.024	Absence
928	0.436	Presence
929	0.345	Presence
930	0.235	Absence
931	0.024	Absence
932	0.305	Presence
933	0.024	Absence
934	0.027	Absence
935	0.433	Presence
936	0.024	Absence
937	0.028	Absence
938	0.024	Absence
939	0.024	Absence
940	0.873	Presence
941	0.024	Absence
942	0.025	Absence
943	0.430	Presence
944	0.263	Presence
945	0.267	Presence
946	0.246	Absence
947	0.486	Presence
948	0.462	Presence
949	0.024	Absence
950	0.024	Absence
951	0.024	Absence
952	0.822	Presence
953	0.769	Presence

954	0.024	Absence
955	0.024	Absence
956	0.164	Absence
957	0.024	Absence
958	0.352	Presence
959	0.319	Presence
960	0.024	Absence
961	0.129	Absence
962	0.043	Absence
963	0.027	Absence
964	0.024	Absence
965	0.024	Absence
966	0.567	Presence
967	0.907	Presence
968	0.116	Absence
969	0.024	Absence
970	0.024	Absence
971	0.527	Presence
972	0.024	Absence
973	0.096	Absence
974	0.026	Absence
975	0.272	Presence
976	0.444	Presence
977	0.032	Absence
978	0.024	Absence
979	0.024	Absence
980	0.036	Absence
981	0.024	Absence
982	0.031	Absence
983	0.024	Absence
984	0.024	Absence
985	0.239	Absence
986	0.160	Absence
987	0.025	Absence
988	0.281	Presence
989	0.588	Presence
990	0.242	Absence
991	0.024	Absence
992	0.406	Presence
993	0.047	Absence
994	0.088	Absence
995	0.159	Absence
996	0.024	Absence
997	0.094	Absence
998	0.024	Absence
999	0.024	Absence
1000	0.215	Absence
1001	0.027	Absence

1002	0.024	Absence
1003	0.156	Absence
1004	0.054	Absence
1005	0.238	Absence
1006	0.024	Absence
1007	0.024	Absence
1008	0.074	Absence
1009	0.024	Absence
1010	0.027	Absence
1011	0.376	Presence
1012	0.046	Absence
1013	0.391	Presence
1014	0.024	Absence
1015	0.435	Presence
1016	0.024	Absence
1017	0.411	Presence
1018	0.024	Absence
1019	0.299	Presence
1020	0.453	Presence
1021	0.208	Absence
1022	0.299	Presence
1023	0.070	Absence
1024	0.024	Absence
1025	0.034	Absence
1026	0.033	Absence
1027	0.171	Absence
1028	0.173	Absence
1029	0.024	Absence
1030	0.028	Absence
1031	0.658	Presence
1032	0.026	Absence
1033	0.037	Absence
1034	0.024	Absence
1035	0.023	Absence
1036	0.387	Presence
1037	0.534	Presence
1038	0.024	Absence
1039	0.103	Absence
1040	0.053	Absence
1041	0.026	Absence
1042	0.024	Absence
1043	0.024	Absence
1044	0.025	Absence
1045	0.131	Absence
1046	0.921	Presence
1047	0.290	Presence
1048	0.345	Presence
1049	0.494	Presence

1050	0.362	Presence
1051	0.372	Presence
1052	0.024	Absence
1053	0.374	Presence
1054	0.024	Absence
1055	0.238	Absence
1056	0.525	Presence
1057	0.023	Absence
1058	0.310	Presence
1059	0.024	Absence
1060	0.127	Absence
1061	0.024	Absence
1062	0.024	Absence
1063	0.421	Presence

Table S3. Population in different potential-risk areas.

Region	Population in potential-risk areas (million, N, %)
**Beilin**	1.15 (14.67)
**Xincheng**	0.99 (12.63)
**Yanta**	0.95 (12.12)
**Weiyang**	0.80 (10.20)
**Chang’an**	0.76 (9.69)
**Lianhu**	0.63 (8.03)
**Lintong**	0.60 (7.65)
**Baqiao**	0.55 (7.02)
**Huyi**	0.51 (6.51)
**Gaolin**	0.29 (3.70)
**Yanliang**	0.24 (3.06)
**Lantian**	0.21 (2.68)
**Zhouzhi**	0.16 (2.04)
**Total**	7.84 (100.00)

Table S4. Data on prevalence of human and animal brucellosis in Inner Mongolia, Gansu, Ningxia, and Shanxi provinces around Xi'an from 2014 to 2019. Data on HB were accessed from the Data-center of China Public Health Science (http://www.phsciencedata.cn/Share/en/index.jsp). Data on animal brucellosis were accessed from the Official Veterinary Bulletin site (http://www.cadc.net.cn/sites/MainSite/tzgg/sygb/). NH, number of human brucellosis; IH, incidence of human brucellosis (1/100,000); NA, number of animal brucellosis.

	**Inner Mongolia**			**Gansu**			**Ningxia**			**Shanxi**		
	**NH**	**IH**	**NA**	**NH**	**IH**	**NA**	**NH**	**IH**	**NA**	**NH**	**IH**	**NA**
**2014**	10135	40.579	16959	1393	5.395	37	2063	31.535	35	8540	23.528	651
**2015**	7238	28.896	20400	2303	8.889	154	2888	43.656	49	6697	19.181	158
**2016**	5970	23.775	7438	1745	6.713	0	2160	32.341	74	4587	12.519	140
**2017**	7347	23.155	6636	1642	6.291	1	1696	25.130	17	3071	8.341	24
**2018**	9708	38.391	9007	1516	5.774	26	1581	23.189	25	2800	7.563	0
**2019**	13780	54.681	2544	1711	6.488	2819	2199	31.962	65	3279	8.818	0

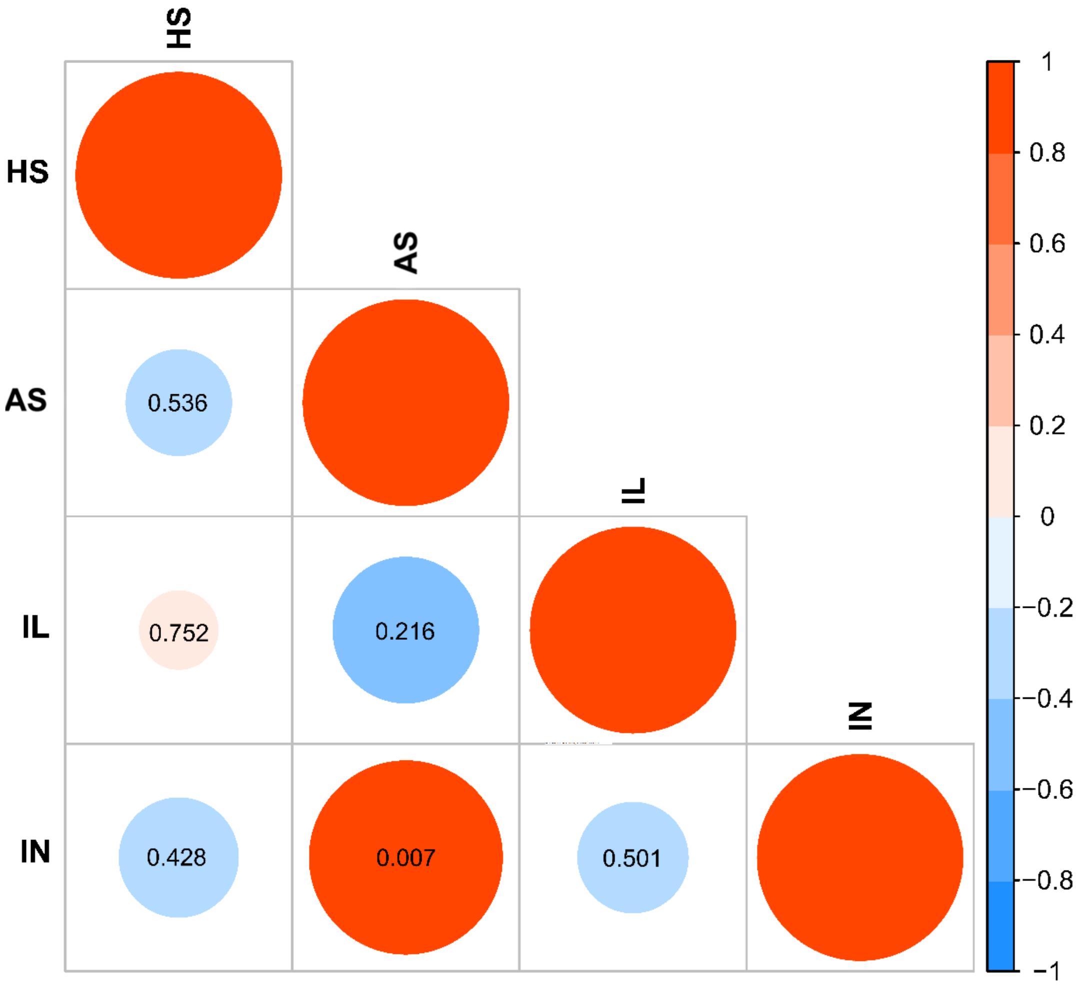

Figure S1. Correlation analysis of seroprevalence and incidence rate in Xi'an, 2014–2021. HS, human seroprevalence rate; AS, animal seroprevalence rate; IL, incidence rate of local cases; IN, incidence rate of nonlocal cases. The numbers in the circles represent the *P*-value of the significance test.
